# Supplementary material for: Splice-Junction-Based Mapping of Alternative Isoforms in the Human Proteome
Source: Cell Rep. Author manuscript; Available in PMC 2020 Jan 15. (PMC6961840; doi:10.1016/j.celrep.2019.11.026)
Supplement: 2 [file NIHMS1546469-supplement-2.zip › DF1/PXD006675/VentricularSeptum/VentricularSeptum_8_RYR2_VLDIANVLFHLEQVEHPQR.pdf]

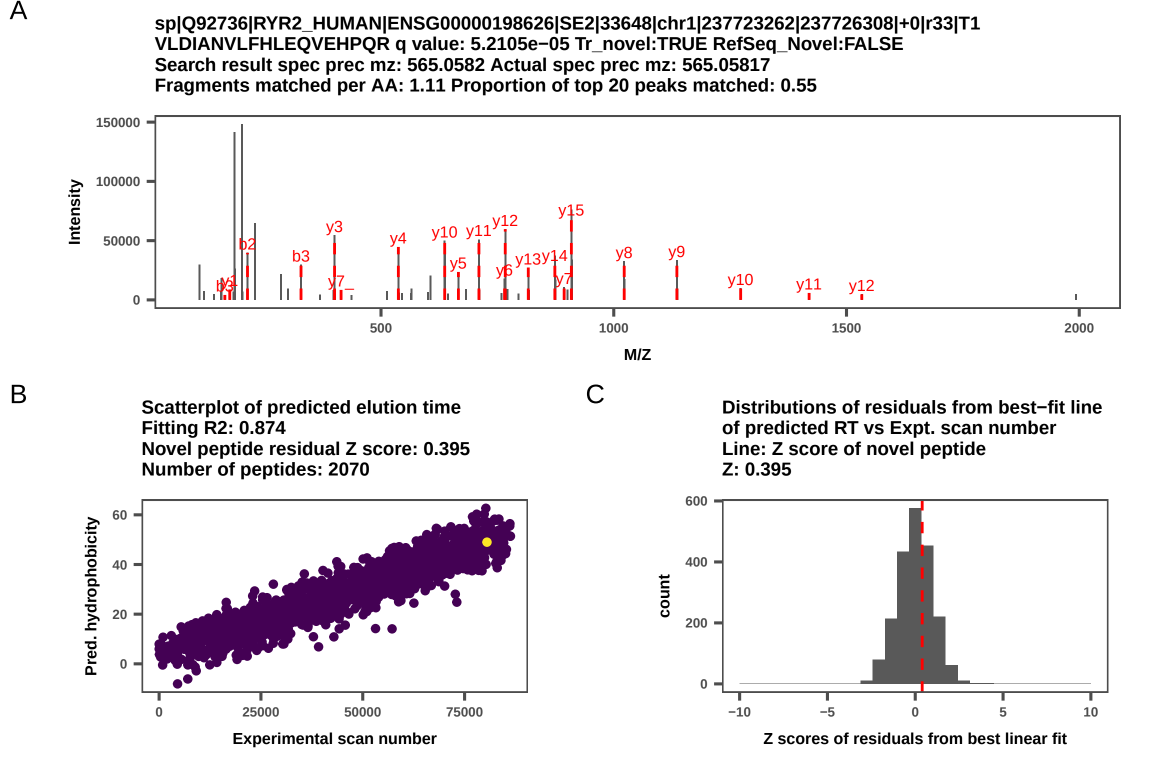
A
sp|Q92736|RYR2_HUMAN|ENSG00000198626|SE2|33648|chr1|237723262|237726308|+0|r33|T1
VLDIANVLFHLEQVEHPQR q value: 5.2105e−05 Tr_novel:TRUE RefSeq_Novel:FALSE
Search result spec prec mz: 565.0582 Actual spec prec mz: 565.05817
Fragments matched per AA: 1.11 Proportion of top 20 peaks matched: 0.55
Intensity
150000
100000
50000
0
b2
b3
y3
y4
y10
y11
y12
y15
y5
y6
y13
y14
y8
y9
y7
y10
y11
y12
500
1000
1500
2000
M/Z
B
Scatterplot of predicted elution time
Fitting R2: 0.874
Novel peptide residual Z score: 0.395
Number of peptides: 2070
Pred. hydrophobicity
60
40
20
0
0
25000
50000
75000
Experimental scan number
C
Distributions of residuals from best−fit line
of predicted RT vs Expt. scan number
Line: Z score of novel peptide
Z: 0.395
count
600
400
200
0
−10
−5
0
5
10
Z scores of residuals from best linear fit
